# Editorial: Innovative approaches to immune tolerance and regulation with gene, cellular, protein, and microbiome based therapeutics

**DOI:** 10.3389/fimmu.2024.1382536

**Published:** 2024-02-28

**Authors:** Amy Rosenberg, Dawn Elaine Smilek

**Affiliations:** ^1^ EpiVax, Inc, Providence, RI, United States; ^2^ Immune Tolerance Network, San Francisco, CA, United States; ^3^ University of California San Francisco, Division of Rheumatology, San Francisco, CA, United States

**Keywords:** cellular, tolerance, Treg - regulatory T cell, GRAIL, microbiome

This Edition includes selected articles focusing on novel approaches to immune tolerance induction. Particularly noteworthy is an article that lays out the foundational basis for establishing immune tolerance in clinical contexts by focusing on boosting the activity of regulatory mechanisms, and specifically of regulatory T cells (Tregs) in a favorable immunologic context: “*Synergistic targeting of immunologic pathways to empower durable tolerance therapies*” by Nepom. Citing studies in Type 1 diabetes mellitus (T1D), Nepom points out that teplizumab (a non-lytic CD3 monoclonal antibody) did not prevent T1D but rather delayed disease onset in some patients by several years, the mechanism of which was induction of exhaustion in CD8+ T effector populations. Nepom emphasized that various approaches for “boosting regulation” in this favorable immunological context could follow for successful “remission induction”. These potential approaches include adoptive Treg therapies, cytokines to boost endogenous Tregs, and presentation of target antigens in a tolerogenic context. Diminishing innate immune responses is also important, given their contribution to adaptive immune responses.

In another noteworthy article “*How GRAIL controls Treg function to maintain self-tolerance*”, Fathman et al focus on Treg function and argue that the defect in self-tolerance in autoimmune disease is due not to numbers but to defects in Treg function pertaining to second messengers downstream of the IL-2R. GRAIL is a ubiquitin E3 ligase whose normal function is to block cullin ring ligase activity, thus inhibiting IL-2R desensitization in Tregs and promoting Treg function. GRAIL’s E3ligase is diminished in Tregs in autoimmune disease and allergic asthma causing degradation of pJAK1, diminished pSTAT5, and decreased downstream transcription of genes critical for Treg function. The authors propose therapeutics to target ubiquitination in Tregs, including neddylation inhibitors. When conjugated with IL-2, neddylation inhibitors demonstrated success in murine autoimmune disease models. Future developments for this agent are highly anticipated.

In “*Biomarkers and mechanisms of tolerance induction in food allergic patients drive new therapeutic approaches*”, Baloh et al discuss novel approaches to tolerance induction in food allergy, including the addition of biologics or other immunomodulators to allergen immunotherapy. In keeping with the overarching strategy, tolerance induction may require complementary immune pathway targeting. A trial of oral peanut therapy combined with omalizumab (mAb to IgE), while not yet demonstrating induction of tolerance, is promising in diminishing adverse events and allowing more rapid oral peanut dosing increases. The selective JAK1 inhibitor abrocitinib is also under study in food allergy, as many pro-allergenic cytokines signal through this common pathway.

In “*Tolerogenic DC generated in vitro using a novel protocol mimicking mucosal tolerance mechanisms represent a potential therapeutic cell platform for induction of immune tolerance*” by Dao Nyesiga et al., CD14+ human monocytes were cultured *in vitro* with compounds of known importance to mucosal immune tolerance including an aryl hydrocarbon receptor agonist, TGF-β, and retinoic acid. The resulting population of DC (ItolDCs) was characterized by decreased expression of HLA-DR and costimulatory molecules, as well as higher expression of tolerance markers including ILT3, CD103 and LAP, and decreased levels of inflammatory cytokines. When Itol DCs were cultured with human PBL, T cell proliferation was diminished and Tregs were boosted compared to other DC populations. The function and stability of Itol DC *in vivo* is yet to be elucidated.

In “*Five-year follow Up of phase I trial of donor derived modified immune cell infusion in kidney transplantation*,” Schaier et al builds on a previous report of a tolerizing protocol for kidney allografts in which patients were infused pre-transplant with donor derived mitomycin-treated PBL. Although the study is small, the results are notable in that treated patients demonstrated clinical benefit: lack of donor specific antibodies (DSA) and no opportunistic infections, while 33% of the retrospective control group had detectable DSA and 53% developed opportunistic infections. Studies undertaken to define the mechanism showed increased IL-10 secreting regulatory B cell populations. Additional studies on the immunologic basis and a prospective study with appropriate comparator groups are needed.

In “*Extracellular vesicles and their cells of origin: Open issues in autoimmune diseases*” by Haghighitalab et al, the advantages of Extracellular Vesicles (ECVs) compared to their intact producer MSC cells in addressing autoimmune diseases are elucidated in a comprehensive review. Key advantages of ECVs include their ability to deeply penetrate tissues and their lower immunogenicity.

There is increasing evidence that the early microbiome influences immunity. “*Effect of early postnatal supplementation of newborns with probiotic strain E. Coli O83: K24:H31 on allergy incidence, dendritic cells and microbiota*” by Sukenikova et al is a 10 year follow up to a study undertaken to investigate pro-biotic supplementation with E. coli O83:K24:H31 in newborns of allergic mothers. Although long term microbiome composition did not differ between E. coli O83:K24:H31treated vs non-treated and allergic vs non-allergic patients, a decreased incidence of allergy in children at age 10 who received the probiotic was observed and associated with increased IL-10 and IFN-γ in sera. These preliminary findings will require verification in additional studies.

A novel class of therapeutics based on endogenous human scaffold proteins engineered to bind to and block disease-associated biological pathways, is the subject of “*Does human homology reduce the potential immunogenicity of non-antibody scaffolds*”. De Groot et al articulate the benefits of in silico epitope mapping tools with human homology analyses to investigate the potential immunogenicity of these novel therapeutics. Modification to the binding region of the fully human scaffold sequence, essential for binding of novel target antigens, gives the highest likelihood of contributing “foreignness” to the scaffold proteins. Prediction and elimination of immunogenic sequences in this setting may optimize the function of these novel therapeutics.

Gene therapy with AAV vectors is complicated by unique immunogenicity challenges. In “*Immunogenicity of AAV Vectors: effects of deamidation on detection of antibody response*” Bing et al address the high rate of spontaneous deamidation of AAV capsids, its effects on AAV T cell immunogenicity, and the issues this generates for evaluation of immunogenicity in AAV clinical trials. Whereas the T cell response in patients may be directed to epitopes modified by deamidation, such responses may not be detected in immunogenicity assays utilizing wild type peptides (WT). The effects of deamidated AAV peptides on detection of preexisting antibodies to AAV9, used to screen patients for clinical studies, was addressed.

Elimination of unwanted immune responses is a serious clinical challenge in allergy, autoimmunity, and transplantation. The approaches outlined in this Edition provide strong rationale for further investigation. As proposed by Nepom, a combination of key synergistic approaches may be required for tolerance induction and consolidation in these clinical indications.

## Author contributions

AR: Conceptualization, Writing – original draft, Writing – review & editing. DS: Conceptualization, Formal analysis, Writing – review & editing.

